# Supplementary material for: A hypothesis on the capacity of plant odorant-binding proteins to bind volatile isoprenoids based on in silico evidences
Source: eLife. 2021 Jun 23;10:e66741. doi: 10.7554/eLife.66741 (PMC8221805; doi:10.7554/eLife.66741)
Supplement: Supplementary file 1. [file elife-66741-supp1.docx]

**Supplementary File 1**

**Plant proteins from sequence databases annotated as “odorant-binding protein”.**

| Accession Number | Protein name | Organism | Sequence length |
| --- | --- | --- | --- |
| A0A1D1ZDX5  (UniProt) ^(a)^ | general odorant-binding protein 56d (OBP56d) | *Anthurium amnicola* | 148aa (fragment) |
| A0A1D1Z329  (UniProt) **^(b)^** | putative odorant-binding protein A10_1 (OBPA10_1) | *Anthurium amnicola* | 149aa (fragment) |
| A0A1D1ZDI0 (UniProt) | putative odorant-binding protein A10_2 (OBPA10_2) | *Anthurium amnicola* | 254aa (fragment) |
| XP_009350846 (NCBI) | general odorant-binding protein 70-like | *Pyrus x bretschneideri* | 170aa (complete) |
| KAF3779823 (UniProt) | putative odorant-binding protein | *Nymphaea thermarum* | 222aa (partial) |

**^(a)^** Annotations in UniProt for this entry revealed that the protein is included into the “Pheromone/general odorant-binding protein superfamily” of the InterPro database (<http://www.ebi.ac.uk/interpro/>).

^(b)^ Annotations in UniProt for this entry revealed that the protein is included into the “Insect odorant-binding protein A10” protein family of the InterPro database.
