# Supplementary material for: A hypothesis on the capacity of plant odorant-binding proteins to bind volatile isoprenoids based on in silico evidences
Source: eLife. 2021 Jun 23;10:e66741. doi: 10.7554/eLife.66741 (PMC8221805; doi:10.7554/eLife.66741)
Supplement: Supplementary file 2. — Only the best ten results are shown. [file elife-66741-supp2.docx]

**Supplementary File 2**

**BLAST results for non-plant proteins with similarity to “general odorant binding protein 56d” from *Anthurium amnicola*. Only the best ten results are shown.**

| Accession Number | Annotation | Organism | Identity  % | Alignment length | E-Value^a^ | Score^a^ |
| --- | --- | --- | --- | --- | --- | --- |
| XP_034232292.1 | general odorant-binding protein 56d-like isoform X1  (151 aa) | *Thrips palmi* | 63.87 | 119 | 4e-51 | 169 |
| XP_026281309.1 | uncharacterized protein LOC113208505  (148 aa) | *Frankliniella occidentalis* | 50.00 | 128 | 5e-28 | 110 |
| KAE8741848.1 | hypothetical protein FOCC_FOCC012596 (190 aa) | *Frankliniella occidentalis* | 50.39 | 127 | 2e-27 | 110 |
| XP_034249409.1 | uncharacterized protein LOC117650253  (149 aa) | *Thrips palmi* | 31.09 | 119 | 3e-11 | 68.2 |
| XP_015835846.1 | PREDICTED: pheromone-binding protein-related protein 6-like  (145 aa) | *Tribolium castaneum* | 30.50 | 141 | 3e-10 | 65.1 |
| EFA04593.1 | odorant binding protein 07 (136 aa) | *Tribolium castaneum* | 29.85 | 134 | 7e-10 | 64.3 |
| XP_019697001.2 | general odorant-binding protein 83a (132 aa) | *Harpegnathos saltator* | 25.37 | 134 | 1e-09 | 63.5 |
| KAF2881145.1 | hypothetical protein ILUMI_25019 (123aa) | *Ignelater luminosus* | 32.23 | 121 | 2e-08 | 60.1 |
| XP_011346593.1 | general odorant-binding protein 83a isoform X1 (133 aa) | *Ooceraea biroi* | 28.57 | 126 | 3e-08 | 59.7 |
| XP_011164418.1 | uncharacterized protein LOC105199157  (131 aa) | *Solenopsis invicta* | 29.01 | 131 | 4e-08 | 59.3 |

^a^ E-value and score are BLAST measures of significance of the sequence similarity observed. Low E-values (< 0.001) and high score indicate very significant results.
