# Supplementary material for: A hypothesis on the capacity of plant odorant-binding proteins to bind volatile isoprenoids based on in silico evidences
Source: eLife. 2021 Jun 23;10:e66741. doi: 10.7554/eLife.66741 (PMC8221805; doi:10.7554/eLife.66741)
Supplement: Supplementary file 3. — Docking simulations by AutoDock suite generated results summarized in the table. For each receptor-ligand simulation, the best 100 conformations are clusterized by AutoDock on the basis of ligand position, and three values are reported for each cluster: the mean binding energy for the clusterized conformations, the best binding energy, the number of conformations in the cluster. An additional parameter computed by AutoDock is the predicted Ki value. We reported the results for the best cluster in terms of energy and population, or in some cases, alternative clusters, with reference to different pockets on the receptor surface. [file elife-66741-supp3.docx]

**Supplementary File 3**

Complete results of docking simulation experiments. When more binding energy and predicted K_i_ values are reported, they refer to alternative binding sites detected.

| Protein | Ligand | Mean  Binding energy (kcal/mol) | Lowest  Binding  Energy  (kcal/mol) | Number in cluster | Predicted Ki | Binding  Pocket ^a^ |
| --- | --- | --- | --- | --- | --- | --- |
| TPL-LIKE | β-caryophyllene | -6.16 | -6.20 | 17 | 28.46 μM | groove (3)-chain A |
|  |  | -6.02 | -6.04 | 31 | 37.50 μM | groove (3)- chain B |
|  | isoprene | -3.39 | -3.46 | 46 | 2.92 mM | Near groove (3) –chain A |
|  |  | -3.41 | -3.45 | 25 | 2.95mM | Near groove (3) –chain B |
|  |  | -2.99 | -3.04 | 16 | 5.94mM | groove (3)- chain A |
|  | α-pinene | -5.06 | -5.13 | 68 | 173.00 μM | groove (3)- chain B |
|  | limonene | -4.76 | -4.78 | 25 | 311.41 μM | Near groove (3) –chain A |
|  |  | -4.76 | -4.78 | 32 | 356.48 μM | LisH-CRA |
|  |  | -4.53 | -4.71 | 33 | 354.67 μM | groove (3)-chain A |
|  | linalool | -3.94 | -4.35 | 16 | 642.79 μM | groove (3)- chain B |
|  |  | -3.76 | -4.13 | 8 | 945.79 μM | groove (3)-chain A |
|  | β-myrcene | -3.95 | -4.19 | 17 | 855.72 μM | groove (3)- chain B |
|  |  | -3.76 | -4.00 | 22 | 1.17 mM | LisH-CRA |
|  |  | -3.77 | -4.03 | 24 | 1.10 mM | groove (3)- chain B |
| 4DSB_A  (ABA RECEPTOR) | ABA | -7.26 | -7.72 | 61 | 2.10 μM | Canonical ABA pocket |
|  | β-caryophyllene | -6.32 | -6.33 | 43 | 22.86 μM | Canonical ABA pocket |
|  | isoprene | -3.35 | -3.42 | 47 | 3.14 mM | Canonical ABA pocket |
|  | α-pinene | -4.69 | -4.69 | 86 | 366.90 μM | Canonical ABA pocket |
|  | limonene | -4.73 | -4.77 | 62 | 319.70 μM | Canonical ABA pocket |
|  | linalool | -4.12 | -4.56 | 30 | 457.50 μM | Canonical ABA pocket |
|  | β-myrcene | -3.84 | -4.12 | 49 | 957.87 μM | Canonical ABA pocket |
| 3EBL_A  (GA RECEPTOR) | GA | -9.31 | -9.43 | 50 | 121.92 nM | Canonical GA pocket |
|  | β-caryophyllene | -6.94 | -7.01 | 83 | 7.30 μM | Canonical GA pocket |
|  | isoprene | -3.78 | -3.81 | 84 | 1.61 mM | Pocket B |
|  | α-pinene | -5.5 | -5.54 | 84 | 87.42 μM | Canonical GA pocket |
|  | limonene | -5.44 | -5.57 | 28 | 82.39 μM | Pocket A |
|  |  | -5.34 | -5.43 | 34 | 104.59 μM | Pocket B |
|  | linalool | -4.69 | -5.16 | 29 | 166.34 μM | Canonical GA pocket |
|  | β-myrcene | -4.10 | -4.30 | 33 | 699.55 μM | Canonical GA pocket |
|  |  | -4.92 | -5.19 | 29 | 157.11 μM | Externally to the canonical GA pocket |
| 3AXY (HD3A) | β-caryophyllene | -6.83 | -6.85 | 93 | 9.57 μM | Pocket A |
|  | isoprene | -3.44 | -3.52 | 86 | 2.64 mM | Pocket B |
|  | α-pinene | -5.46 | -5.52 | 87 | 90.24 μM | Pocket B |
|  | limonene | -4.99 | -5.02 | 41 | 209.73 μM | Pocket A |
|  |  | -4.9 | -4.93 | 15 | 242.72 μM | Pocket B |
|  | linalool | -4.96 | -5.23 | 23 | 147.07 μM | Pocket B |
|  |  | -4.72 | -4.93 | 36 | 241.47 μM | Pocket A |
|  | β-myrcene | -4.26 | -4.61 | 32 | 415.20 μM | Pocket B |
|  |  | -4.29 | -4.52 | 49 | 488.40 μM | Pocket A |
| 1WKP (FLOWERING LOCUS T) | β-caryophyllene | -6.65 | -6.66 | 62 | 13.22 μM | Pocket A |
|  |  | -6.19 | -6.20 | 29 | 28.48 μM | Pocket B |
|  | isoprene | -3.15 | -3.19 | 78 | 4.61 mM | Pocket C |
|  | α-pinene | -5.14 | -5.26 | 7 | 139.67 μM | Pocket C |
|  |  | -5.23 | -5.24 | 77 | 143.49 μM | Pocket B |
|  | limonene | -5.07 | -5.10 | 47 | 181.16 μM | Pocket A |
|  |  | -4.82 | -4.95 | 20 | 234.45μM | Pocket C |
|  |  | -4.77 | -4.78 | 17 | 312.82 μM | Pocket B |
|  | linalool | -4.79 | -4.98 | 27 | 224.96 μM | Pocket A |
|  |  | -4.64 | -4.90 | 18 | 256.30 μM | Pocket B |
|  | β-myrcene | -4.26 | -4.54 | 23 | 473.68 μM | Pocket C |
|  |  | -4.25 | -4.49 | 22 (+17) | 513.11 μM | Pocket A |
|  |  | -4.09 | -4.25 | 21 | 770.36 μM | Pocket B |
| 1WKO (TFL1) | β-caryophyllene | -7.15 | -7.17 | 38 | 5.57 μM | Pocket A |
|  |  | -6.91 | -6.91 | 59 | 120.59 μM | Pocket B |
|  | isoprene | -3.70 | -3.74 | 63 | 1.83 mM | Pocket C |
|  | α-pinene | -5.30 | -5.35 | 73 | 120.59 μM | Pocket A |
|  |  | -4.84 | -4.86 | 14 | 272.98 μM | Pocket B |
|  | limonene | -5.53 | -5.61 | 44 | 77.40 μM | Pocket C |
|  |  | -5.12 | -5.17 | 19 | 161.41 μM | Pocket A |
|  |  | -4.88 | -4.91 | 22 | 251.03 μM | Pocket B |
|  | linalool | -4.98 | -5.46 | 16 (+5+11+4+1) | 99.74 μM | Pocket A |
|  |  | -4.68 | -4.91 | 22 (+16) | 251 μM | Pocket B |
|  | β-myrcene | -4.38 | -4.58 | 29 | 442.69 μM | Pocket A |
|  |  | -4.00 | -4.15 | 31 | 911.60 μM | Pocket B |
| COI  (partial JA receptor) | JA-Ile | -4.54 | -4.54 | 1 | 65.30 μM | Canonical JA pocket |
|  | β-caryophyllene | -6.05 | -6.11 | 10 | 33.18 μM | Canonical JA pocket |
|  | isoprene | -2.78 | -2.80 | 2 | 8.90 mM | Canonical JA pocket |
|  | α-pinene | -5.23 | -5.30 | 16 | 130.15 μM | Canonical JA pocket |
|  |  | -5.00 | -5.03 | 46 | 206.18 μM | Pocket A |
|  | limonene | -4.79 | -4.97 | 25 (+19) | 228.74 μM | Pocket A |
|  |  | -4.68 | -4.74 | 21 | 333.01 μM | Canonical JA pocket |
|  | linalool | -4.21 | -4.85 | 24 | 278.52 μM | Pocket B |
|  |  | -3.91 | -4.15 | 7 | 903.47 μM | Canonical JA pocket |
|  | β-myrcene | -4.16 | -4.42 | 36 | 571.25 μM | Pocket A |
|  |  | -3.82 | -4.04 | 18 | 1.09 mM | Canonical JA pocket |
| COI & JAZ  (JA receptor complete) | JA-Ile | not available | | | | |
|  | β-caryophyllene | -6.57 | -6.58 | 9 | 15.04 μM | Canonical JA pocket |
|  |  | -6.61 | -6.64 | 38 | 13.50 μM | Pocket A |
|  | isoprene | -2.82 | -2.84 | 2 | 8.29 mM | Canonical JA pocket |
|  | α-pinene | -5.92 | -6.02 | 59 | 38.55 μM | Pocket B |
|  |  | -5.39 | -5.49 | 14 (+1) | 94.57 μM | Canonical JA pocket |
|  |  | -5.11 | -5.12 | 15 | 176.86 μM | Pocket A |
|  | limonene | -5.11 | -5.25 | 38 | 321.92 μM | Canonical JA pocket |
|  | linalool | -4.49 | -4.76 | 25 | 142.75 μM | Canonical JA pocket |
|  | β-myrcene | -4.41 | -4.62 | 40 | 321.92 μM | Canonical JA pocket |
|  |  | -4.23 | -4.53 | 28 | 408.11 μM | Pocket A |
| SABP2  (SA enzyme) | SA | -5.37 | -5.47 | 34 | 97.25 μM | Canonical SA binding pocket |
|  | β-caryophyllene | -6.73 | -6.75 | 3 | 11.21 μM | Canonical SA binding pocket |
|  |  | -5.71 | -5.71 | 77 | 64.92 μM | Pocket B |
|  | isoprene | -3.25 | -3.33 | 3 | 3.65 mM | Canonical SA binding pocket |
|  |  | -3.24 | -3.26 | 91 | 4.06 mM | Externally to the canonical SA binding Pocket |
|  | α-pinene | -6.03 | -6.04 | 57 | 37.39 μM | Canonical SA binding pocket |
|  |  | -4.82 | -4.82 | 33 | 290.70 μM | Pocket B |
|  | limonene | -6.12 | -6.14 | 78 | 31.49 μM | Canonical SA binding pocket |
|  |  | -4.32 | -4.37 | 16 | 629.66 μM | Pocket B |
|  | linalool | -4.7 | -5.02 | 17 (+7+2+1) | 208.22 μM | Canonical SA binding pocket |
|  |  | -3.86 | -4.17 | 28 | 2.01 mM | Pocket B |
|  | β-myrcene | -5.14 | -5.43 | 57 (+14) | 104.29 μM | Canonical SA binding pocket |
|  |  | -3.75 | -4.07 | 27 | 1.04 mM | Pocket B |

^a^ Binding pocket column describes the site where the ligand binds. In most cases, the name of the site is the description of a specific protein region. In some cases, pocket A/B/C refer to different sites where the ligands bind.
