# Supplementary material for: A hypothesis on the capacity of plant odorant-binding proteins to bind volatile isoprenoids based on in silico evidences
Source: eLife. 2021 Jun 23;10:e66741. doi: 10.7554/eLife.66741 (PMC8221805; doi:10.7554/eLife.66741)
Supplement: Supplementary file 4. [file elife-66741-supp4.docx]

Supplementary File 4

List of 432 protein sequences selected as OBP from animal sources, used for searching public database for similar plant proteins.

>sp|O02372|OB76A_DROME General odorant-binding protein lush OS=Drosophila melanogaster OX=7227 GN=lush PE=1 SV=1

>sp|Q9NY56|OBP2A_HUMAN Odorant-binding protein 2a OS=Homo sapiens OX=9606 GN=OBP2A PE=1 SV=1

>sp|P54191|OB69A_DROME General odorant-binding protein 69a OS=Drosophila melanogaster OX=7227 GN=Obp69a PE=1 SV=2

>sp|Q18434|GPA17_CAEEL Guanine nucleotide-binding protein alpha-17 subunit OS=Caenorhabditis elegans OX=6239 GN=odr-3 PE=1 SV=1

>sp|Q7TQQ0|O1509_MOUSE Olfactory receptor 1509 OS=Mus musculus OX=10090 GN=Olfr1509 PE=1 SV=1

>sp|Q9VNB5|ORCO_DROME Odorant receptor coreceptor OS=Drosophila melanogaster OX=7227 GN=Orco PE=1 SV=2

>sp|B1Q257|GCY10_CAEEL Receptor-type guanylate cyclase gcy-10 OS=Caenorhabditis elegans OX=6239 GN=odr-1 PE=1 SV=2

>sp|Q18807|ODR10_CAEEL Serpentine receptor class r-10 OS=Caenorhabditis elegans OX=6239 GN=odr-10 PE=1 SV=3

>sp|Q9PW88|GPC6A_CARAU G-protein coupled receptor family C group 6 member A OS=Carassius auratus OX=7957 GN=gprc6a PE=1 SV=1

>sp|Q9V8Y9|OB56H_DROME General odorant-binding protein 56h OS=Drosophila melanogaster OX=7227 GN=Obp56h PE=2 SV=2

>sp|P81909|OR22A_DROME Odorant receptor 22a OS=Drosophila melanogaster OX=7227 GN=Or22a PE=1 SV=1

>sp|P81910|OR22B_DROME Odorant receptor 22b OS=Drosophila melanogaster OX=7227 GN=Or22b PE=1 SV=3

>sp|Q16EI9|OR4_AEDAE Odorant receptor 4 OS=Aedes aegypti OX=7159 GN=GPROR4 PE=2 SV=1

>sp|P41933|ODR7_CAEEL Nuclear hormone receptor family member odr-7 OS=Caenorhabditis elegans OX=6239 GN=odr-7 PE=1 SV=1

>sp|E2BJ30|ORCO_HARSA Odorant receptor coreceptor OS=Harpegnathos saltator OX=610380 GN=Orco PE=1 SV=1

>sp|Q7QCC7|ORCO_ANOGA Odorant receptor coreceptor OS=Anopheles gambiae OX=7165 GN=Orco PE=1 SV=3

>sp|Q60895|OL150_MOUSE Olfactory receptor 150 OS=Mus musculus OX=10090 GN=Olfr150 PE=3 SV=3

>sp|A0A026W182|ORCO_OOCBI Odorant receptor coreceptor OS=Ooceraea biroi OX=2015173 GN=Orco PE=1 SV=1

>sp|Q8WP90|PBGP9_SOLIN Pheromone-binding protein Gp-9 OS=Solenopsis invicta OX=13686 GN=Gp-9 PE=1 SV=1

>sp|Q86FX7|GPA17_CAEBR Guanine nucleotide-binding protein alpha-17 subunit OS=Caenorhabditis briggsae OX=6238 GN=odr-3 PE=3 SV=1

>sp|Q5EP01|PBGP9_SOLMG Pheromone-binding protein Gp-9 OS=Solenopsis megergates OX=310439 GN=Gp-9 PE=3 SV=1

>sp|Q9D3H2|OBP1A_MOUSE Odorant-binding protein 1a OS=Mus musculus OX=10090 GN=Obp1a PE=1 SV=2

>sp|Q9VT92|OR67D_DROME Odorant receptor 67d OS=Drosophila melanogaster OX=7227 GN=Or67d PE=2 SV=3

>sp|Q178U6|ORCO_AEDAE Odorant receptor coreceptor OS=Aedes aegypti OX=7159 GN=SGPRor7 PE=1 SV=1

>sp|P23275|OLF15_MOUSE Olfactory receptor 15 OS=Mus musculus OX=10090 GN=Olfr15 PE=2 SV=4

>sp|P08937|OBP_RAT Odorant-binding protein OS=Rattus norvegicus OX=10116 GN=Obp1f PE=1 SV=1

>sp|Q9NPH6|OBP2B_HUMAN Odorant-binding protein 2b OS=Homo sapiens OX=9606 GN=OBP2B PE=2 SV=1

>sp|P81917|OR43A_DROME Odorant receptor 43a OS=Drosophila melanogaster OX=7227 GN=Or43a PE=1 SV=2

>sp|Q9VHQ7|OR85B_DROME Odorant receptor 85b OS=Drosophila melanogaster OX=7227 GN=Or85b PE=1 SV=2

>sp|P81921|OR47A_DROME Odorant receptor 47a OS=Drosophila melanogaster OX=7227 GN=Or47a PE=1 SV=1

>sp|A2AEP0|OBP1B_MOUSE Odorant-binding protein 1b OS=Mus musculus OX=10090 GN=Obp1b PE=1 SV=1

>sp|P81912|OR23A_DROME Odorant receptor 23a OS=Drosophila melanogaster OX=7227 GN=Or23a PE=2 SV=1

>sp|Q9V3N2|OR46B_DROME Odorant receptor 46a, isoform B OS=Drosophila melanogaster OX=7227 GN=Or46a PE=2 SV=2

>sp|P81919|OR46A_DROME Odorant receptor 46a, isoform A OS=Drosophila melanogaster OX=7227 GN=Or46a PE=2 SV=4

>sp|Q5U9X3|GPC6A_DANRE G-protein coupled receptor family C group 6 member A OS=Danio rerio OX=7955 GN=gprc6a PE=1 SV=1

>sp|A9LKE4|PBGP9_SOLQU Pheromone-binding protein Gp-9 OS=Solenopsis quinquecuspis OX=176596 GN=Gp-9 PE=3 SV=2

>sp|Q9VNB3|OR83A_DROME Odorant receptor 83a OS=Drosophila melanogaster OX=7227 GN=Or83a PE=3 SV=2

>sp|P81918|OR43B_DROME Odorant receptor 43b OS=Drosophila melanogaster OX=7227 GN=Or43b PE=2 SV=3

>sp|Q5ENZ1|PBGP9_SOLSV Pheromone-binding protein Gp-9 OS=Solenopsis saevissima OX=176597 GN=Gp-9 PE=3 SV=1

>sp|P81245|OBP_PIG Odorant-binding protein OS=Sus scrofa OX=9823 PE=1 SV=1

>sp|P54193|OB83A_DROME General odorant-binding protein 83a OS=Drosophila melanogaster OX=7227 GN=Obp83a PE=1 SV=1

>sp|P81923|OR59A_DROME Odorant receptor 59a OS=Drosophila melanogaster OX=7227 GN=Or59a PE=2 SV=2

>sp|A9LKF0|PBGP9_SOLRI Pheromone-binding protein Gp-9 OS=Solenopsis richteri OX=30203 GN=Gp-9 PE=3 SV=2

>sp|Q8WTE7|OR1_ANOGA Odorant receptor Or1 OS=Anopheles gambiae OX=7165 GN=OR1 PE=2 SV=1

>sp|Q8WTE6|OR2_ANOGA Odorant receptor Or2 OS=Anopheles gambiae OX=7165 GN=OR2 PE=2 SV=1

>sp|Q86BF9|OB59A_DROME Odorant-binding protein 59a OS=Drosophila melanogaster OX=7227 GN=Obp59a PE=1 SV=1

>sp|O46077|OR2A_DROME Odorant receptor 2a OS=Drosophila melanogaster OX=7227 GN=Or2a PE=2 SV=2

>sp|P81915|OR33B_DROME Odorant receptor 33b OS=Drosophila melanogaster OX=7227 GN=Or33b PE=2 SV=1

>sp|Q9VAI6|OB99B_DROME General odorant-binding protein 99b OS=Drosophila melanogaster OX=7227 GN=Obp99b PE=2 SV=1

>sp|Q9W1P8|OR59B_DROME Odorant receptor 59b OS=Drosophila melanogaster OX=7227 GN=Or59b PE=1 SV=1

>sp|Q5EP12|PBGP9_SOLGE Pheromone-binding protein Gp-9 OS=Solenopsis geminata OX=121131 GN=Gp-9 PE=3 SV=1

>sp|Q9VT08|OR67A_DROME Odorant receptor 67a OS=Drosophila melanogaster OX=7227 GN=Or67a PE=1 SV=2

>sp|Q9V3Q2|OR35A_DROME Odorant receptor 35a OS=Drosophila melanogaster OX=7227 GN=Or35a PE=1 SV=3

>sp|Q9V9I2|OR42A_DROME Odorant receptor 42a OS=Drosophila melanogaster OX=7227 GN=Or42a PE=2 SV=3

>sp|P51875|GNAO_CAEEL Guanine nucleotide-binding protein G(o) subunit alpha OS=Caenorhabditis elegans OX=6239 GN=goa-1 PE=1 SV=3

>sp|Q9W5G6|OR1A_DROME Odorant receptor 1a OS=Drosophila melanogaster OX=7227 GN=Or1a PE=2 SV=2

>sp|P24394|IL4RA_HUMAN Interleukin-4 receptor subunit alpha OS=Homo sapiens OX=9606 GN=IL4R PE=1 SV=1

>sp|P81914|OR33A_DROME Odorant receptor 33a OS=Drosophila melanogaster OX=7227 GN=Or33a PE=2 SV=1

>sp|P81916|OR33C_DROME Odorant receptor 33c OS=Drosophila melanogaster OX=7227 GN=Or33c PE=2 SV=1

>sp|A8XC92|ODR10_CAEBR Serpentine receptor class r-10 OS=Caenorhabditis briggsae OX=6238 GN=odr-10 PE=3 SV=2

>sp|Q9V8Y2|OB56A_DROME General odorant-binding protein 56a OS=Drosophila melanogaster OX=7227 GN=Obp56a PE=2 SV=1

>sp|Q8VEZ0|OL480_MOUSE Olfactory receptor 480 OS=Mus musculus OX=10090 GN=Olfr480 PE=3 SV=1

>sp|P81924|OR85E_DROME Putative odorant receptor 85e OS=Drosophila melanogaster OX=7227 GN=Or85e PE=2 SV=3

>sp|Q9V9I4|OR42B_DROME Odorant receptor 42b OS=Drosophila melanogaster OX=7227 GN=Or42b PE=2 SV=2

>sp|P81922|OR47B_DROME Odorant receptor 47b OS=Drosophila melanogaster OX=7227 GN=Or47b PE=2 SV=2

>sp|P82986|OR82A_DROME Odorant receptor 82a OS=Drosophila melanogaster OX=7227 GN=Or82a PE=2 SV=1

>sp|P54192|OB19D_DROME General odorant-binding protein 19d OS=Drosophila melanogaster OX=7227 GN=Obp19d PE=2 SV=2

>sp|Q9V6A9|OR49A_DROME Odorant receptor 49a OS=Drosophila melanogaster OX=7227 GN=Or49a PE=3 SV=3

>sp|Q9V6H2|OR49B_DROME Odorant receptor 49b OS=Drosophila melanogaster OX=7227 GN=Or49b PE=2 SV=1

>sp|P34985|OL143_MOUSE Olfactory receptor 143 OS=Mus musculus OX=10090 GN=Olfr143 PE=2 SV=2

>sp|Q9V8Y7|OR56A_DROME Odorant receptor 56a OS=Drosophila melanogaster OX=7227 GN=Or56a PE=2 SV=2

>sp|Q9VHE6|OR85F_DROME Odorant receptor 85f OS=Drosophila melanogaster OX=7227 GN=Or85f PE=2 SV=1

>sp|Q9VHS4|OR85A_DROME Odorant receptor 85a OS=Drosophila melanogaster OX=7227 GN=Or85a PE=2 SV=1

>sp|Q9W3I5|OR7A_DROME Odorant receptor 7a OS=Drosophila melanogaster OX=7227 GN=Or7a PE=2 SV=1

>sp|P81911|OR22C_DROME Odorant receptor 22c OS=Drosophila melanogaster OX=7227 GN=Or22c PE=2 SV=2

>sp|P81913|OR24A_DROME Odorant receptor 24a OS=Drosophila melanogaster OX=7227 GN=Or24a PE=2 SV=4

>sp|Q9VYZ1|OR10A_DROME Odorant receptor 10a OS=Drosophila melanogaster OX=7227 GN=Or10a PE=2 SV=1

>sp|Q9VAZ3|OR98A_DROME Odorant receptor 98a OS=Drosophila melanogaster OX=7227 GN=Or98a PE=2 SV=2

>sp|Q9VAJ4|OB99A_DROME General odorant-binding protein 99a OS=Drosophila melanogaster OX=7227 GN=Obp99a PE=2 SV=2

>sp|P34170|OBP2_BOMMO General odorant-binding protein 2 OS=Bombyx mori OX=7091 PE=1 SV=2

>sp|Q60882|OL145_MOUSE Olfactory receptor 145 OS=Mus musculus OX=10090 GN=Olfr145 PE=3 SV=2

>sp|P31419|OBP2_MANSE General odorant-binding protein 2 OS=Manduca sexta OX=7130 GN=GOBP2 PE=1 SV=1

>sp|P31418|OBP1_MANSE General odorant-binding protein 1 OS=Manduca sexta OX=7130 GN=GOBP1 PE=1 SV=1

>sp|C0HJA7|OBP1_RABIT Odorant-binding protein 1 (Fragment) OS=Oryctolagus cuniculus OX=9986 PE=1 SV=1

>sp|O76584|GPA11_CAEEL Guanine nucleotide-binding protein alpha-11 subunit OS=Caenorhabditis elegans OX=6239 GN=gpa-11 PE=2 SV=2

>sp|Q9VR94|OB19A_DROME General odorant-binding protein 19a OS=Drosophila melanogaster OX=7227 GN=Obp19a PE=2 SV=2

>sp|P54194|OB84A_DROME General odorant-binding protein 84a OS=Drosophila melanogaster OX=7227 GN=Obp84a PE=2 SV=1

>sp|Q8MMF9|OB57D_DROME General odorant-binding protein 57d OS=Drosophila melanogaster OX=7227 GN=Obp57d PE=2 SV=2

>sp|P34171|OBP1_BOMMO General odorant-binding protein 1 OS=Bombyx mori OX=7091 PE=1 SV=2

>sp|Q9W2U9|OR9A_DROME Odorant receptor 9a OS=Drosophila melanogaster OX=7227 GN=Or9a PE=2 SV=1

>sp|Q9VT90|OR67C_DROME Odorant receptor 67c OS=Drosophila melanogaster OX=7227 GN=Or67c PE=2 SV=2

>sp|A9LKF6|PBGP9_SOLMC Pheromone-binding protein Gp-9 OS=Solenopsis macdonaghi OX=176595 GN=Gp-9 PE=3 SV=2

>sp|Q9I816|OR19A_DROME Odorant receptor 19a OS=Drosophila melanogaster OX=7227 GN=Or19a PE=2 SV=2

>sp|Q9VHQ6|OR85C_DROME Odorant receptor 85c OS=Drosophila melanogaster OX=7227 GN=Or85c PE=3 SV=2

>sp|Q9VCS9|OR94A_DROME Odorant receptor 94a OS=Drosophila melanogaster OX=7227 GN=Or94a PE=3 SV=1

>sp|Q9VXL0|OR13A_DROME Odorant receptor 13a OS=Drosophila melanogaster OX=7227 GN=Or13a PE=2 SV=2

>sp|P07435|OBP_BOVIN Odorant-binding protein OS=Bos taurus OX=9913 PE=1 SV=2

>sp|P38406|GNAL_RAT Guanine nucleotide-binding protein G(olf) subunit alpha OS=Rattus norvegicus OX=10116 GN=Gnal PE=2 SV=2

>sp|Q60884|OL146_MOUSE Olfactory receptor 146 OS=Mus musculus OX=10090 GN=Olfr146 PE=2 SV=2

>sp|P82982|OR65A_DROME Odorant receptor 65a OS=Drosophila melanogaster OX=7227 GN=Or65a PE=2 SV=1

>sp|Q60886|OL147_MOUSE Olfactory receptor 147 OS=Mus musculus OX=10090 GN=Olfr147 PE=3 SV=2

>sp|Q5EP06|PBGP9_SOLEE Pheromone-binding protein Gp-9 OS=Solenopsis electra OX=227486 GN=Gp-9 PE=3 SV=1

>sp|Q5EP05|PBGP9_SOLDA Pheromone-binding protein Gp-9 OS=Solenopsis daguerrei OX=310437 GN=Gp-9 PE=3 SV=1

>sp|Q9VFN2|OR88A_DROME Odorant receptor 88a OS=Drosophila melanogaster OX=7227 GN=Or88a PE=2 SV=2

>sp|Q5EP07|PBGP9_SOLPU Pheromone-binding protein Gp-9 OS=Solenopsis pusillignis OX=310436 GN=Gp-9 PE=3 SV=1

>sp|Q9VU27|OR69A_DROME Putative odorant receptor 69a, isoform A OS=Drosophila melanogaster OX=7227 GN=Or69a PE=2 SV=2

>sp|Q60893|OL151_MOUSE Olfactory receptor 151 OS=Mus musculus OX=10090 GN=Olfr151 PE=2 SV=2

>sp|P82985|OR69B_DROME Putative odorant receptor 69a, isoform B OS=Drosophila melanogaster OX=7227 GN=Or69a PE=2 SV=1

>sp|Q8K1H9|OBP2A_MOUSE Odorant-binding protein 2a OS=Mus musculus OX=10090 GN=Obp2a PE=2 SV=1

>sp|Q23970|PBP6_DROME Pheromone-binding protein-related protein 6 OS=Drosophila melanogaster OX=7227 GN=Obp83b PE=1 SV=2

>sp|C0HJA9|OBP3_RABIT Odorant-binding protein 3 (Fragment) OS=Oryctolagus cuniculus OX=9986 PE=1 SV=1

>sp|Q9V568|OR45A_DROME Odorant receptor 45a OS=Drosophila melanogaster OX=7227 GN=Or45a PE=3 SV=3

>sp|Q9VT20|OR67B_DROME Odorant receptor 67b OS=Drosophila melanogaster OX=7227 GN=Or67b PE=2 SV=2

>sp|Q9VVF3|OR74A_DROME Odorant receptor 74a OS=Drosophila melanogaster OX=7227 GN=Or74a PE=3 SV=1

>sp|Q60894|OLF12_MOUSE Olfactory receptor 12 OS=Mus musculus OX=10090 GN=Olfr12 PE=3 SV=2

>sp|Q9VLE5|OR30A_DROME Odorant receptor 30a OS=Drosophila melanogaster OX=7227 GN=Or30a PE=3 SV=4

>sp|Q9V589|OR45B_DROME Odorant receptor 45b OS=Drosophila melanogaster OX=7227 GN=Or45b PE=3 SV=1

>sp|Q9VZW8|OR63A_DROME Odorant receptor 63a OS=Drosophila melanogaster OX=7227 GN=Or63a PE=3 SV=2

>sp|Q9VCS8|OR94B_DROME Odorant receptor 94b OS=Drosophila melanogaster OX=7227 GN=Or94b PE=3 SV=1

>sp|P34983|O1537_MOUSE Olfactory receptor 1537 OS=Mus musculus OX=10090 GN=Olfr1537 PE=3 SV=2

>sp|P54195|OB28A_DROME General odorant-binding protein 28a OS=Drosophila melanogaster OX=7227 GN=Obp28a PE=2 SV=2

>sp|Q8SY61|OB56D_DROME General odorant-binding protein 56d OS=Drosophila melanogaster OX=7227 GN=Obp56d PE=1 SV=2

>sp|Q60885|OLF9_MOUSE Olfactory receptor 9 OS=Mus musculus OX=10090 GN=Olfr9 PE=3 SV=2

>sp|P59827|BPIB4_HUMAN BPI fold-containing family B member 4 OS=Homo sapiens OX=9606 GN=BPIFB4 PE=2 SV=2

>sp|Q9VNK9|OR83C_DROME Putative odorant receptor 83c OS=Drosophila melanogaster OX=7227 GN=Or83c PE=2 SV=2

>sp|C0HJA6|OBP2_RABIT Odorant-binding protein 2 (Fragment) OS=Oryctolagus cuniculus OX=9986 PE=1 SV=1

>sp|Q86DA5|SARM1_CAEEL NAD(+) hydrolase tir-1 OS=Caenorhabditis elegans OX=6239 GN=tir-1 PE=1 SV=1

>sp|Q17075|OBP2_ANTPE General odorant-binding protein 2 OS=Antheraea pernyi OX=7119 PE=2 SV=1

>sp|Q9H902|REEP1_HUMAN Receptor expression-enhancing protein 1 OS=Homo sapiens OX=9606 GN=REEP1 PE=1 SV=1

>sp|P59826|BPIB3_HUMAN BPI fold-containing family B member 3 OS=Homo sapiens OX=9606 GN=BPIFB3 PE=1 SV=2

>sp|Q9W1P7|OR59C_DROME Putative odorant receptor 59c OS=Drosophila melanogaster OX=7227 GN=Or59c PE=2 SV=1

>sp|P81647|OBP1_HYSCR Odorant-binding protein 1 (Fragment) OS=Hystrix cristata OX=10137 PE=1 SV=1

>sp|P81648|OBP2_HYSCR Odorant-binding protein 2 (Fragment) OS=Hystrix cristata OX=10137 PE=1 SV=1

>sp|O62305|KCC2D_CAEEL Calcium/calmodulin-dependent protein kinase type II OS=Caenorhabditis elegans OX=6239 GN=unc-43 PE=1 SV=2

>sp|Q9BRK0|REEP2_HUMAN Receptor expression-enhancing protein 2 OS=Homo sapiens OX=9606 GN=REEP2 PE=1 SV=2

>sp|Q21029|NSY1_CAEEL Mitogen-activated protein kinase kinase kinase nsy-1 OS=Caenorhabditis elegans OX=6239 GN=nsy-1 PE=1 SV=4

>sp|S5ZYD3|OBP_PHOSU Odorant-binding protein (Fragment) OS=Phodopus sungorus OX=10044 GN=OBP PE=1 SV=1

>sp|Q9V938|OB57E_DROME General odorant-binding protein 57e OS=Drosophila melanogaster OX=7227 GN=Obp57e PE=3 SV=4

>sp|P34982|OR1D2_HUMAN Olfactory receptor 1D2 OS=Homo sapiens OX=9606 GN=OR1D2 PE=1 SV=2

>sp|Q9VUK5|OR71A_DROME Putative odorant receptor 71a OS=Drosophila melanogaster OX=7227 GN=Or71a PE=2 SV=4

>sp|Q9VHQ2|OR85D_DROME Putative odorant receptor 85d OS=Drosophila melanogaster OX=7227 GN=Or85d PE=2 SV=1

>sp|Q95VF0|PBP2_EPIPO Pheromone-binding protein 2 OS=Epiphyas postvittana OX=65032 GN=PBP PE=1 SV=1

>sp|Q50EX6|ODO1_PETHY Protein ODORANT1 OS=Petunia hybrida OX=4102 GN=ODO1 PE=2 SV=1

>sp|Q16S34|OBP45_AEDAE General odorant-binding protein 45 OS=Aedes aegypti OX=7159 GN=Obp45 PE=1 SV=1

>sp|Q95VP3|GOBP1_EPIPO General odorant-binding protein 1 OS=Epiphyas postvittana OX=65032 PE=1 SV=1

>sp|Q95VP2|GOBP2_EPIPO General odorant-binding protein 2 OS=Epiphyas postvittana OX=65032 PE=1 SV=1

>sp|P51839|GUC2D_RAT Guanylate cyclase 2D OS=Rattus norvegicus OX=10116 GN=Gucy2d PE=1 SV=2

>sp|Q5TYJ0|OBP71_ANOGA General odorant-binding protein 71 OS=Anopheles gambiae OX=7165 GN=Obp71 PE=3 SV=4

>sp|O70191|ATF5_MOUSE Cyclic AMP-dependent transcription factor ATF-5 OS=Mus musculus OX=10090 GN=Atf5 PE=1 SV=2

>sp|P63018|HSP7C_RAT Heat shock cognate 71 kDa protein OS=Rattus norvegicus OX=10116 GN=Hspa8 PE=1 SV=1

>sp|O76360|EGL4_CAEEL cGMP-dependent protein kinase egl-4 OS=Caenorhabditis elegans OX=6239 GN=egl-4 PE=1 SV=2

>sp|P07154|CATL1_RAT Cathepsin L1 OS=Rattus norvegicus OX=10116 GN=Ctsl PE=1 SV=2

>sp|G5EDF7|SEK1_CAEEL Dual specificity mitogen-activated protein kinase kinase sek-1 OS=Caenorhabditis elegans OX=6239 GN=sek-1 PE=1 SV=1

>sp|P21932|ADCY3_RAT Adenylate cyclase type 3 OS=Rattus norvegicus OX=10116 GN=Adcy3 PE=1 SV=1

>sp|Q9NFU0|FMR1_DROME Synaptic functional regulator FMR1 OS=Drosophila melanogaster OX=7227 GN=Fmr1 PE=1 SV=1

>sp|P46580|ANM5_CAEEL Protein arginine N-methyltransferase 5 OS=Caenorhabditis elegans OX=6239 GN=prmt-5 PE=1 SV=2

>sp|Q8I4N4|DAF11_CAEEL Receptor-type guanylate cyclase daf-11 OS=Caenorhabditis elegans OX=6239 GN=daf-11 PE=1 SV=3

>sp|Q8VGS3|O1019_MOUSE Olfactory receptor 1019 OS=Mus musculus OX=10090 GN=Olfr1019 PE=1 SV=1

>sp|Q96DX8|RTP4_HUMAN Receptor-transporting protein 4 OS=Homo sapiens OX=9606 GN=RTP4 PE=1 SV=3

>sp|Q8CFW1|ANO2_MOUSE Anoctamin-2 OS=Mus musculus OX=10090 GN=Ano2 PE=1 SV=2

>sp|Q9NQ90|ANO2_HUMAN Anoctamin-2 OS=Homo sapiens OX=9606 GN=ANO2 PE=1 SV=2

>sp|Q5EP08|PBGP9_SOLXY Pheromone-binding protein Gp-9 OS=Solenopsis xyloni OX=310435 GN=Gp-9 PE=3 SV=1

>sp|Q5EP17|PBGP9_SOLS1 Pheromone-binding protein Gp-9 OS=Solenopsis sp. (strain B0-178) OX=310431 GN=Gp-9 PE=3 SV=1

>sp|Q5EP14|PBGP9_SOLTR Pheromone-binding protein Gp-9 OS=Solenopsis tridens OX=310434 GN=Gp-9 PE=3 SV=1

>sp|Q5EP16|PBGP9_SOLNG Pheromone-binding protein Gp-9 OS=Solenopsis nigella gensterblumi OX=310432 GN=Gp-9 PE=3 SV=1

>sp|Q5ENZ7|PBGP9_SOLSB Pheromone-binding protein Gp-9 OS=Solenopsis sp. (strain B0-151) OX=310440 GN=Gp-9 PE=3 SV=1

>sp|Q5ENZ6|PBGP9_SOLS0 Pheromone-binding protein Gp-9 OS=Solenopsis sp. (strain B0-153) OX=310441 GN=Gp-9 PE=3 SV=1

>sp|Q5QGT7|RTP2_HUMAN Receptor-transporting protein 2 OS=Homo sapiens OX=9606 GN=RTP2 PE=1 SV=1

>sp|Q9VDM1|OR92A_DROME Putative odorant receptor 92a OS=Drosophila melanogaster OX=7227 GN=Or92a PE=3 SV=3

>sp|Q5EP15|PBGP9_SOLSU Pheromone-binding protein Gp-9 OS=Solenopsis substituta OX=310433 GN=Gp-9 PE=3 SV=1

>sp|Q5EP04|PBGP9_SOLSJ Pheromone-binding protein Gp-9 OS=Solenopsis n. sp. (strain JP-2002) OX=310438 GN=Gp-9 PE=3 SV=1

>sp|Q9H6H4|REEP4_HUMAN Receptor expression-enhancing protein 4 OS=Homo sapiens OX=9606 GN=REEP4 PE=1 SV=1

>sp|P82983|OR65B_DROME Putative odorant receptor 65b OS=Drosophila melanogaster OX=7227 GN=Or65b PE=3 SV=2

>sp|Q8IRZ5|OR19B_DROME Putative odorant receptor 19b OS=Drosophila melanogaster OX=7227 GN=Or19b PE=3 SV=1

>sp|P82984|OR65C_DROME Putative odorant receptor 65c OS=Drosophila melanogaster OX=7227 GN=Or65c PE=3 SV=2

>sp|Q7K4Y6|DAT_DROME Sodium-dependent dopamine transporter OS=Drosophila melanogaster OX=7227 GN=DAT PE=1 SV=1

>sp|Q9VAW0|OR98B_DROME Putative odorant receptor 98b OS=Drosophila melanogaster OX=7227 GN=Or98b PE=3 SV=3

>sp|P87508|OBP1_ANTPE General odorant-binding protein 1 OS=Antheraea pernyi OX=7119 PE=2 SV=1

>sp|Q9XZS8|ATF3_DROME Activating transcription factor 3 OS=Drosophila melanogaster OX=7227 GN=Atf3 PE=1 SV=1

>sp|Q9V931|OB57C_DROME General odorant-binding protein 57c OS=Drosophila melanogaster OX=7227 GN=Obp57c PE=2 SV=1

>sp|Q8MKJ4|OB57B_DROME General odorant-binding protein 57b OS=Drosophila melanogaster OX=7227 GN=Obp57b PE=2 SV=1

>sp|Q8MKK0|OB57A_DROME General odorant-binding protein 57a OS=Drosophila melanogaster OX=7227 GN=Obp57a PE=2 SV=1

>sp|P04905|GSTM1_RAT Glutathione S-transferase Mu 1 OS=Rattus norvegicus OX=10116 GN=Gstm1 PE=1 SV=2

>sp|Q64625|GPX6_RAT Glutathione peroxidase 6 OS=Rattus norvegicus OX=10116 GN=Gpx6 PE=2 SV=1

>sp|P08010|GSTM2_RAT Glutathione S-transferase Mu 2 OS=Rattus norvegicus OX=10116 GN=Gstm2 PE=1 SV=2

>sp|O60266|ADCY3_HUMAN Adenylate cyclase type 3 OS=Homo sapiens OX=9606 GN=ADCY3 PE=1 SV=3

>sp|Q8VHH7|ADCY3_MOUSE Adenylate cyclase type 3 OS=Mus musculus OX=10090 GN=Adcy3 PE=1 SV=2

>sp|P70377|FGF13_MOUSE Fibroblast growth factor 13 OS=Mus musculus OX=10090 GN=Fgf13 PE=1 SV=2

>sp|P50281|MMP14_HUMAN Matrix metalloproteinase-14 OS=Homo sapiens OX=9606 GN=MMP14 PE=1 SV=3

>sp|Q00195|CNGA2_RAT Cyclic nucleotide-gated olfactory channel OS=Rattus norvegicus OX=10116 GN=Cnga2 PE=1 SV=1

>sp|A0A0U1RPR8|GUC2D_MOUSE Guanylate cyclase D OS=Mus musculus OX=10090 GN=Gucy2d PE=2 SV=1

>sp|Q9ERW3|FGF13_RAT Fibroblast growth factor 13 OS=Rattus norvegicus OX=10116 GN=Fgf13 PE=1 SV=2

>sp|P00786|CATH_RAT Pro-cathepsin H OS=Rattus norvegicus OX=10116 GN=Ctsh PE=1 SV=1

>sp|Q64338|PDE1C_MOUSE Calcium/calmodulin-dependent 3',5'-cyclic nucleotide phosphodiesterase 1C OS=Mus musculus OX=10090 GN=Pde1c PE=1 SV=2

>sp|Q27377|OB10_DROME Putative odorant-binding protein A10 OS=Drosophila melanogaster OX=7227 GN=a10 PE=2 SV=2

>sp|P53690|MMP14_MOUSE Matrix metalloproteinase-14 OS=Mus musculus OX=10090 GN=Mmp14 PE=2 SV=3

>sp|O60911|CATL2_HUMAN Cathepsin L2 OS=Homo sapiens OX=9606 GN=CTSV PE=1 SV=2

>sp|A7UR17|OBP72_ANOGA General odorant-binding protein 72 (Fragment) OS=Anopheles gambiae OX=7165 GN=Obp72 PE=3 SV=1

>sp|Q8BGH4|REEP1_MOUSE Receptor expression-enhancing protein 1 OS=Mus musculus OX=10090 GN=Reep1 PE=1 SV=1

>sp|Q10739|MMP14_RAT Matrix metalloproteinase-14 OS=Rattus norvegicus OX=10116 GN=Mmp14 PE=2 SV=2

>sp|Q63257|IL4RA_RAT Interleukin-4 receptor subunit alpha OS=Rattus norvegicus OX=10116 GN=Il4r PE=2 SV=2

>sp|Q64359|CNGA4_RAT Cyclic nucleotide-gated cation channel alpha-4 OS=Rattus norvegicus OX=10116 GN=Cnga4 PE=2 SV=1

>sp|Q02955|IL1R1_RAT Interleukin-1 receptor type 1 OS=Rattus norvegicus OX=10116 GN=Il1r1 PE=2 SV=1

>sp|Q63421|PDE1C_RAT Calcium/calmodulin-dependent 3',5'-cyclic nucleotide phosphodiesterase 1C OS=Rattus norvegicus OX=10116 GN=Pde1c PE=1 SV=1

>sp|Q14123|PDE1C_HUMAN Calcium/calmodulin-dependent 3',5'-cyclic nucleotide phosphodiesterase 1C OS=Homo sapiens OX=9606 GN=PDE1C PE=1 SV=1

>sp|Q16280|CNGA2_HUMAN Cyclic nucleotide-gated olfactory channel OS=Homo sapiens OX=9606 GN=CNGA2 PE=2 SV=2

>sp|O35548|MMP16_RAT Matrix metalloproteinase-16 OS=Rattus norvegicus OX=10116 GN=Mmp16 PE=2 SV=1

>sp|Q9VET0|NPF_DROME Neuropeptide F OS=Drosophila melanogaster OX=7227 GN=NPF PE=1 SV=1

>sp|Q8NGS4|O13F1_HUMAN Olfactory receptor 13F1 OS=Homo sapiens OX=9606 GN=OR13F1 PE=2 SV=1

>sp|Q9VVL1|IR75A_DROME Ionotropic receptor 75a OS=Drosophila melanogaster OX=7227 GN=Ir75a PE=1 SV=2

>sp|Q7TN78|ACSM4_RAT Acyl-coenzyme A synthetase ACSM4, mitochondrial OS=Rattus norvegicus OX=10116 GN=Acsm4 PE=1 SV=1

>sp|Q62398|CNGA2_MOUSE Cyclic nucleotide-gated olfactory channel OS=Mus musculus OX=10090 GN=Cnga2 PE=2 SV=2

>sp|Q7Q5L4|OBP70_ANOGA General odorant-binding protein 70 OS=Anopheles gambiae OX=7165 GN=Obp70 PE=3 SV=2

>sp|A0NBD9|OBP66_ANOGA General odorant-binding protein 66 OS=Anopheles gambiae OX=7165 GN=Obp66 PE=3 SV=1

>sp|F5HK49|OBP69_ANOGA General odorant-binding protein 69 OS=Anopheles gambiae OX=7165 GN=Obp69 PE=3 SV=1

>sp|Q5TXN1|OBP67_ANOGA General odorant-binding protein 67 OS=Anopheles gambiae OX=7165 GN=Obp67 PE=3 SV=2

>sp|Q8IV77|CNGA4_HUMAN Cyclic nucleotide-gated cation channel alpha-4 OS=Homo sapiens OX=9606 GN=CNGA4 PE=1 SV=3

>sp|A0NAZ8|OBP68_ANOGA General odorant-binding protein 68 OS=Anopheles gambiae OX=7165 GN=Obp68 PE=3 SV=1

>sp|O44857|NEPL2_CAEEL Neprilysin-2 OS=Caenorhabditis elegans OX=6239 GN=nep-2 PE=1 SV=2

>sp|Q9NVN3|RIC8B_HUMAN Synembryn-B OS=Homo sapiens OX=9606 GN=RIC8B PE=1 SV=2

>sp|Q8NGH5|O56A1_HUMAN Olfactory receptor 56A1 OS=Homo sapiens OX=9606 GN=OR56A1 PE=2 SV=3

>sp|Q8NGH8|O56A4_HUMAN Olfactory receptor 56A4 OS=Homo sapiens OX=9606 GN=OR56A4 PE=2 SV=2

>sp|Q95VE9|PBP1_EPIPO Pheromone-binding protein 1 OS=Epiphyas postvittana OX=65032 GN=PBP PE=1 SV=1

>sp|Q8NG75|OR5T1_HUMAN Olfactory receptor 5T1 OS=Homo sapiens OX=9606 GN=OR5T1 PE=2 SV=1

>sp|P51485|ARRB_CAEEL Beta-arrestin arr-1 OS=Caenorhabditis elegans OX=6239 GN=arr-1 PE=1 SV=2

>sp|Q5QNP2|TA13C_DANRE Trace amine-associated receptor 13c OS=Danio rerio OX=7955 GN=taar13c PE=1 SV=1

>sp|Q9X839|CYC2_STRCO Germacradienol/geosmin synthase OS=Streptomyces coelicolor (strain ATCC BAA-471 / A3(2) / M145) OX=100226 GN=cyc2 PE=1 SV=3

>sp|Q27288|OBP2_HELVI General odorant-binding protein 2 OS=Heliothis virescens OX=7102 PE=2 SV=1

>sp|Q27226|OBP1_HELVI General odorant-binding protein 1 OS=Heliothis virescens OX=7102 PE=2 SV=1

>sp|P20797|PBP_ANTPO Pheromone-binding protein OS=Antheraea polyphemus OX=7120 PE=1 SV=1

>sp|Q8WZ84|OR8D1_HUMAN Olfactory receptor 8D1 OS=Homo sapiens OX=9606 GN=OR8D1 PE=2 SV=1

>sp|P34172|OBP_HYACE General odorant-binding protein (Fragment) OS=Hyalophora cecropia OX=7123 PE=1 SV=1

>sp|P34169|OBP_ANTPO General odorant-binding protein (Fragment) OS=Antheraea polyphemus OX=7120 PE=1 SV=1

>sp|P34173|OBP_LYMDI General odorant-binding protein (Fragment) OS=Lymantria dispar OX=13123 PE=1 SV=1

>sp|P0C7T3|O56A5_HUMAN Olfactory receptor 56A5 OS=Homo sapiens OX=9606 GN=OR56A5 PE=3 SV=1

>sp|Q05701|BPIB3_RAT BPI fold-containing family B member 3 OS=Rattus norvegicus OX=10116 GN=Bpifb3 PE=2 SV=1

>sp|Q27597|NCPR_DROME NADPH--cytochrome P450 reductase OS=Drosophila melanogaster OX=7227 GN=Cpr PE=2 SV=2

>sp|P34174|PBP_BOMMO Pheromone-binding protein OS=Bombyx mori OX=7091 PE=1 SV=2

>sp|A9LKE6|PBGP9_SOLSX Pheromone-binding protein Gp-9 OS=Solenopsis sp. (strain X) OX=326717 GN=Gp-9 PE=3 SV=1

>sp|Q7KF17|PBGP9_SOLAU Pheromone-binding protein Gp-9 OS=Solenopsis aurea OX=176591 GN=Gp-9 PE=3 SV=1

>sp|Q8WP92|PBGP9_SOLAM Pheromone-binding protein Gp-9 OS=Solenopsis amblychila OX=176590 GN=Gp-9 PE=3 SV=1

>sp|Q8WRP6|PBGP9_SOLGI Pheromone-binding protein Gp-9 OS=Solenopsis globularia littoralis OX=176593 GN=Gp-9 PE=3 SV=1

>sp|Q8WRQ4|PBGP9_SOLIT Pheromone-binding protein Gp-9 OS=Solenopsis interrupta OX=176594 GN=Gp-9 PE=3 SV=1

>sp|P18959|PBP_MANSE Pheromone-binding protein OS=Manduca sexta OX=7130 PE=1 SV=1

>sp|Q8VCD6|REEP2_MOUSE Receptor expression-enhancing protein 2 OS=Mus musculus OX=10090 GN=Reep2 PE=1 SV=2

>sp|Q8WZ94|OR5P3_HUMAN Olfactory receptor 5P3 OS=Homo sapiens OX=9606 GN=OR5P3 PE=2 SV=1

>sp|Q3UW12|CNGA4_MOUSE Cyclic nucleotide-gated cation channel alpha-4 OS=Mus musculus OX=10090 GN=Cnga4 PE=2 SV=1

>sp|Q8NGV6|OR5H6_HUMAN Olfactory receptor 5H6 OS=Homo sapiens OX=9606 GN=OR5H6 PE=2 SV=2

>sp|P54185|OBA5_DROME Putative odorant-binding protein A5 OS=Drosophila melanogaster OX=7227 GN=a5 PE=2 SV=2

>sp|Q9BQQ7|RTP3_HUMAN Receptor-transporting protein 3 OS=Homo sapiens OX=9606 GN=RTP3 PE=1 SV=1

>sp|Q9GZM6|OR8D2_HUMAN Olfactory receptor 8D2 OS=Homo sapiens OX=9606 GN=OR8D2 PE=2 SV=1

>sp|A2BGH0|BPIB4_MOUSE BPI fold-containing family B member 4 OS=Mus musculus OX=10090 GN=Bpifb4 PE=2 SV=1

>sp|Q27018|B2_TENMO B2 protein (Fragment) OS=Tenebrio molitor OX=7067 PE=2 SV=1

>sp|Q28718|CNGA2_RABIT Cyclic nucleotide-gated olfactory channel OS=Oryctolagus cuniculus OX=9986 GN=CNGA2 PE=2 SV=1

>sp|P18153|ALL2_AEDAE 37 kDa salivary gland allergen Aed a 2 OS=Aedes aegypti OX=7159 GN=D7 PE=1 SV=2

>sp|Q8VFX2|O1444_MOUSE Olfactory receptor 1444 OS=Mus musculus OX=10090 GN=Olfr1444 PE=3 SV=1

>sp|Q8VFL5|O1030_MOUSE Olfactory receptor 1030 OS=Mus musculus OX=10090 GN=Olfr1030 PE=3 SV=1

>sp|Q8VGR8|O1052_MOUSE Olfactory receptor 1052 OS=Mus musculus OX=10090 GN=Olfr1052 PE=2 SV=1

>sp|Q8VF76|OL998_MOUSE Olfactory receptor 998 OS=Mus musculus OX=10090 GN=Olfr998 PE=3 SV=2

>sp|P37072|OLF6_CHICK Olfactory receptor-like protein COR6 OS=Gallus gallus OX=9031 GN=COR6 PE=3 SV=2

>sp|A6NMS3|OR5K4_HUMAN Olfactory receptor 5K4 OS=Homo sapiens OX=9606 GN=OR5K4 PE=3 SV=1

>sp|Q8NGL2|OR5L1_HUMAN Olfactory receptor 5L1 OS=Homo sapiens OX=9606 GN=OR5L1 PE=2 SV=1

>sp|Q8NGL0|OR5L2_HUMAN Olfactory receptor 5L2 OS=Homo sapiens OX=9606 GN=OR5L2 PE=2 SV=1

>sp|Q8NGP4|OR5M3_HUMAN Olfactory receptor 5M3 OS=Homo sapiens OX=9606 GN=OR5M3 PE=2 SV=2

>sp|Q8NGQ5|OR9Q1_HUMAN Olfactory receptor 9Q1 OS=Homo sapiens OX=9606 GN=OR9Q1 PE=2 SV=1

>sp|Q8NGG6|OR8BC_HUMAN Olfactory receptor 8B12 OS=Homo sapiens OX=9606 GN=OR8B12 PE=2 SV=1

>sp|Q8NG78|OR8G5_HUMAN Olfactory receptor 8G5 OS=Homo sapiens OX=9606 GN=OR8G5 PE=2 SV=2

>sp|Q8NH89|O5AK3_HUMAN Putative olfactory receptor 5AK3 OS=Homo sapiens OX=9606 GN=OR5AK3P PE=5 SV=1

>sp|Q8VGI4|OL476_MOUSE Olfactory receptor 476 OS=Mus musculus OX=10090 GN=Olfr476 PE=3 SV=1

>sp|P37068|OLF2_CHICK Olfactory receptor-like protein COR2 OS=Gallus gallus OX=9031 GN=COR2 PE=3 SV=1

>sp|Q8NGP8|OR5M1_HUMAN Olfactory receptor 5M1 OS=Homo sapiens OX=9606 GN=OR5M1 PE=3 SV=1

>sp|P0DMU2|OR83P_HUMAN Putative olfactory receptor 8G3 pseudogene OS=Homo sapiens OX=9606 GN=OR8G3P PE=5 SV=1

>sp|Q8NGF7|OR5BH_HUMAN Olfactory receptor 5B17 OS=Homo sapiens OX=9606 GN=OR5B17 PE=3 SV=1

>sp|Q8NGP2|OR8J1_HUMAN Olfactory receptor 8J1 OS=Homo sapiens OX=9606 GN=OR8J1 PE=2 SV=2

>sp|Q8NGW1|OR6B3_HUMAN Olfactory receptor 6B3 OS=Homo sapiens OX=9606 GN=OR6B3 PE=3 SV=1

>sp|Q80ZU7|BPIB3_MOUSE BPI fold-containing family B member 3 OS=Mus musculus OX=10090 GN=Bpifb3 PE=2 SV=2

>sp|A6NDH6|O5H15_HUMAN Olfactory receptor 5H15 OS=Homo sapiens OX=9606 GN=OR5H15 PE=3 SV=1

>sp|Q8NHC7|O14CZ_HUMAN Olfactory receptor 14C36 OS=Homo sapiens OX=9606 GN=OR14C36 PE=3 SV=1

>sp|Q9Z1V0|OLF49_MOUSE Olfactory receptor 49 OS=Mus musculus OX=10090 GN=Olfr49 PE=2 SV=1

>sp|Q8VFD1|OL492_MOUSE Olfactory receptor 492 OS=Mus musculus OX=10090 GN=Olfr492 PE=3 SV=1

>sp|Q8NH10|OR8U1_HUMAN Olfactory receptor 8U1 OS=Homo sapiens OX=9606 GN=OR8U1 PE=3 SV=1

>sp|Q05704|BPIB4_RAT BPI fold-containing family B member 4 OS=Rattus norvegicus OX=10116 GN=Bpifb4 PE=2 SV=2

>sp|Q9F1Y6|MIBS_STRCO 2-methylisoborneol synthase OS=Streptomyces coelicolor (strain ATCC BAA-471 / A3(2) / M145) OX=100226 GN=SCO7700 PE=1 SV=1

>sp|Q6IF36|O8G2P_HUMAN Putative olfactory receptor 8G2 OS=Homo sapiens OX=9606 GN=OR8G2P PE=5 SV=1

>sp|Q8VG02|OL488_MOUSE Olfactory receptor 488 OS=Mus musculus OX=10090 GN=Olfr488 PE=3 SV=1

>sp|Q8NGI8|O5AN1_HUMAN Olfactory receptor 5AN1 OS=Homo sapiens OX=9606 GN=OR5AN1 PE=2 SV=1

>sp|Q8NGC0|O5AU1_HUMAN Olfactory receptor 5AU1 OS=Homo sapiens OX=9606 GN=OR5AU1 PE=2 SV=2

>sp|Q8NGQ1|OR9G4_HUMAN Olfactory receptor 9G4 OS=Homo sapiens OX=9606 GN=OR9G4 PE=3 SV=2

>sp|Q17078|PBP2_ANTPE Pheromone-binding protein 2 OS=Antheraea pernyi OX=7119 PE=2 SV=1

>sp|Q9ER80|RTP4_MOUSE Receptor-transporting protein 4 OS=Mus musculus OX=10090 GN=Rtp4 PE=1 SV=1

>sp|O95221|OR5F1_HUMAN Olfactory receptor 5F1 OS=Homo sapiens OX=9606 GN=OR5F1 PE=2 SV=2

>sp|P0DN80|OR5H8_HUMAN Olfactory receptor 5H8 OS=Homo sapiens OX=9606 GN=OR5H8 PE=3 SV=1

>sp|Q96RC9|OR8B4_HUMAN Olfactory receptor 8B4 OS=Homo sapiens OX=9606 GN=OR8B4 PE=2 SV=2

>sp|Q8NGG1|OR8J2_HUMAN Olfactory receptor 8J2 OS=Homo sapiens OX=9606 GN=OR8J2 PE=3 SV=2

>sp|Q8K072|REEP4_MOUSE Receptor expression-enhancing protein 4 OS=Mus musculus OX=10090 GN=Reep4 PE=1 SV=1

>sp|Q80ZG0|RIC8B_RAT Synembryn-B OS=Rattus norvegicus OX=10116 GN=Ric8b PE=1 SV=1

>sp|Q8VFK2|O1002_MOUSE Olfactory receptor 1002 OS=Mus musculus OX=10090 GN=Olfr1002 PE=3 SV=2

>sp|P81285|OBPA_MAMBR Antennal odorant-binding protein (Fragment) OS=Mamestra brassicae OX=55057 PE=1 SV=1

>sp|P37070|OLF4_CHICK Olfactory receptor-like protein COR4 OS=Gallus gallus OX=9031 GN=COR4 PE=3 SV=1

>sp|Q13606|OR5I1_HUMAN Olfactory receptor 5I1 OS=Homo sapiens OX=9606 GN=OR5I1 PE=2 SV=1

>sp|Q8NH85|OR5R1_HUMAN Olfactory receptor 5R1 OS=Homo sapiens OX=9606 GN=OR5R1 PE=3 SV=1

>sp|Q99KK1|REEP3_MOUSE Receptor expression-enhancing protein 3 OS=Mus musculus OX=10090 GN=Reep3 PE=1 SV=1

>sp|Q8NGL4|OR5DD_HUMAN Olfactory receptor 5D13 OS=Homo sapiens OX=9606 GN=OR5D13 PE=3 SV=2

>sp|Q8NH51|OR8K3_HUMAN Olfactory receptor 8K3 OS=Homo sapiens OX=9606 GN=OR8K3 PE=3 SV=1

>sp|Q03041|CNGA2_BOVIN Cyclic nucleotide-gated olfactory channel OS=Bos taurus OX=9913 GN=CNGA2 PE=1 SV=1

>sp|Q8NGI9|OR5A2_HUMAN Olfactory receptor 5A2 OS=Homo sapiens OX=9606 GN=OR5A2 PE=2 SV=1

>sp|Q8N0Y5|OR8I2_HUMAN Olfactory receptor 8I2 OS=Homo sapiens OX=9606 GN=OR8I2 PE=2 SV=1

>sp|Q6IFH4|OR6B2_HUMAN Olfactory receptor 6B2 OS=Homo sapiens OX=9606 GN=OR6B2 PE=2 SV=2

>sp|P0C628|O5AC1_HUMAN Olfactory receptor 5AC1 OS=Homo sapiens OX=9606 GN=OR5AC1 PE=3 SV=1

>sp|Q8VFD0|OL486_MOUSE Olfactory receptor 486 OS=Mus musculus OX=10090 GN=Olfr486 PE=3 SV=1

>sp|P0C7N5|OR8U9_HUMAN Olfactory receptor 8U9 OS=Homo sapiens OX=9606 GN=OR8U9 PE=3 SV=1

>sp|Q8NGV7|OR5H2_HUMAN Olfactory receptor 5H2 OS=Homo sapiens OX=9606 GN=OR5H2 PE=3 SV=3

>sp|Q8NH48|OR5B3_HUMAN Olfactory receptor 5B3 OS=Homo sapiens OX=9606 GN=OR5B3 PE=3 SV=1

>sp|Q8NGG4|OR8H1_HUMAN Olfactory receptor 8H1 OS=Homo sapiens OX=9606 GN=OR8H1 PE=2 SV=1

>sp|Q96R54|O14A2_HUMAN Olfactory receptor 14A2 OS=Homo sapiens OX=9606 GN=OR14A2 PE=3 SV=2

>sp|Q8VEX6|OL187_MOUSE Olfactory receptor 187 OS=Mus musculus OX=10090 GN=Olfr187 PE=2 SV=1

>sp|A6NET4|OR5K3_HUMAN Olfactory receptor 5K3 OS=Homo sapiens OX=9606 GN=OR5K3 PE=3 SV=1

>sp|Q8NH50|OR8K5_HUMAN Olfactory receptor 8K5 OS=Homo sapiens OX=9606 GN=OR8K5 PE=3 SV=1

>sp|Q8NGR1|O13A1_HUMAN Olfactory receptor 13A1 OS=Homo sapiens OX=9606 GN=OR13A1 PE=2 SV=2

>sp|Q8VG04|OL478_MOUSE Olfactory receptor 478 OS=Mus musculus OX=10090 GN=Olfr478 PE=3 SV=1

>sp|Q60880|OL141_MOUSE Olfactory receptor 141 OS=Mus musculus OX=10090 GN=Olfr141 PE=3 SV=2

>sp|Q7TS48|OL180_MOUSE Olfactory receptor Olfr180 OS=Mus musculus OX=10090 GN=Olfr180 PE=3 SV=1

>sp|Q98913|OLF8_CHICK Olfactory receptor-like protein COR8 (Fragment) OS=Gallus gallus OX=9031 GN=COR8 PE=3 SV=1

>sp|Q8NGK9|OR5DG_HUMAN Olfactory receptor 5D16 OS=Homo sapiens OX=9606 GN=OR5D16 PE=3 SV=1

>sp|Q15617|OR8G1_HUMAN Olfactory receptor 8G1 OS=Homo sapiens OX=9606 GN=OR8G1 PE=2 SV=2

>sp|P34176|PBP1_LYMDI Pheromone-binding protein 1 (Fragment) OS=Lymantria dispar OX=13123 PE=1 SV=1

>sp|Q9UGF5|O14J1_HUMAN Olfactory receptor 14J1 OS=Homo sapiens OX=9606 GN=OR14J1 PE=2 SV=1

>sp|Q8VF12|OL495_MOUSE Olfactory receptor 495 OS=Mus musculus OX=10090 GN=Olfr495 PE=3 SV=1

>sp|A6ND48|O14I1_HUMAN Olfactory receptor 14I1 OS=Homo sapiens OX=9606 GN=OR14I1 PE=1 SV=1

>sp|Q9TU99|OR1G1_PANTR Olfactory receptor 1G1 OS=Pan troglodytes OX=9598 GN=OR1G1 PE=3 SV=1

>sp|Q8WZ92|OR5P2_HUMAN Olfactory receptor 5P2 OS=Homo sapiens OX=9606 GN=OR5P2 PE=2 SV=1

>sp|Q8NH69|OR5W2_HUMAN Olfactory receptor 5W2 OS=Homo sapiens OX=9606 GN=OR5W2 PE=3 SV=1

>sp|Q8NGF6|O10W1_HUMAN Olfactory receptor 10W1 OS=Homo sapiens OX=9606 GN=OR10W1 PE=2 SV=1

>sp|Q8VGR9|O1044_MOUSE Olfactory receptor 1044 OS=Mus musculus OX=10090 GN=Olfr1044 PE=3 SV=1

>sp|Q8VG03|OL482_MOUSE Olfactory receptor 482 OS=Mus musculus OX=10090 GN=Olfr482 PE=3 SV=1

>sp|Q8VG05|OL483_MOUSE Olfactory receptor 483 OS=Mus musculus OX=10090 GN=Olfr483 PE=3 SV=1

>sp|Q8VG42|OL508_MOUSE Olfactory receptor 508 OS=Mus musculus OX=10090 GN=Olfr508 PE=3 SV=1

>sp|A6NKK0|OR5H1_HUMAN Olfactory receptor 5H1 OS=Homo sapiens OX=9606 GN=OR5H1 PE=1 SV=1

>sp|P0C617|O5AL1_HUMAN Olfactory receptor 5AL1 OS=Homo sapiens OX=9606 GN=OR5AL1 PE=3 SV=2

>sp|Q8NHC5|O14AG_HUMAN Olfactory receptor 14A16 OS=Homo sapiens OX=9606 GN=OR14A16 PE=3 SV=1

>sp|Q8VFK7|O1020_MOUSE Olfactory receptor 1020 OS=Mus musculus OX=10090 GN=Olfr1020 PE=3 SV=1

>sp|A6NHG9|O5H14_HUMAN Olfactory receptor 5H14 OS=Homo sapiens OX=9606 GN=OR5H14 PE=2 SV=1

>sp|Q8NHB7|OR5K1_HUMAN Olfactory receptor 5K1 OS=Homo sapiens OX=9606 GN=OR5K1 PE=2 SV=2

>sp|Q8VFK1|O1009_MOUSE Olfactory receptor 1009 OS=Mus musculus OX=10090 GN=Olfr1009 PE=3 SV=1

>sp|Q80ZI2|RTP2_MOUSE Receptor-transporting protein 2 OS=Mus musculus OX=10090 GN=Rtp2 PE=1 SV=1

>sp|Q8NH90|O5AK2_HUMAN Olfactory receptor 5AK2 OS=Homo sapiens OX=9606 GN=OR5AK2 PE=2 SV=1

>sp|Q8NGQ6|OR9I1_HUMAN Olfactory receptor 9I1 OS=Homo sapiens OX=9606 GN=OR9I1 PE=3 SV=1

>sp|Q96RB7|OR5MB_HUMAN Olfactory receptor 5M11 OS=Homo sapiens OX=9606 GN=OR5M11 PE=2 SV=2

>sp|Q8N146|OR8H3_HUMAN Olfactory receptor 8H3 OS=Homo sapiens OX=9606 GN=OR8H3 PE=3 SV=1

>sp|P34177|PBP2_LYMDI Pheromone-binding protein 2 (Fragment) OS=Lymantria dispar OX=13123 PE=1 SV=1

>sp|Q8VFL9|O1086_MOUSE Olfactory receptor 1086 OS=Mus musculus OX=10090 GN=Olfr1086 PE=3 SV=1

>sp|Q8VEW5|OL493_MOUSE Olfactory receptor 493 OS=Mus musculus OX=10090 GN=Olfr493 PE=3 SV=1

>sp|Q8VGQ7|OL181_MOUSE Olfactory receptor 181 OS=Mus musculus OX=10090 GN=Olfr181 PE=2 SV=3

>sp|Q8NGL3|OR5DE_HUMAN Olfactory receptor 5D14 OS=Homo sapiens OX=9606 GN=OR5D14 PE=3 SV=1

>sp|Q6NUK4|REEP3_HUMAN Receptor expression-enhancing protein 3 OS=Homo sapiens OX=9606 GN=REEP3 PE=1 SV=1

>sp|Q5PQN0|NCALD_RAT Neurocalcin-delta OS=Rattus norvegicus OX=10116 GN=Ncald PE=1 SV=3

>sp|Q9QY00|OL154_MOUSE Olfactory receptor 154 OS=Mus musculus OX=10090 GN=Olfr154 PE=1 SV=2

>sp|Q9TU89|OR3A1_GORGO Olfactory receptor 3A1 OS=Gorilla gorilla gorilla OX=9595 GN=OR3A1 PE=3 SV=1

>sp|Q8NGP3|OR5M9_HUMAN Olfactory receptor 5M9 OS=Homo sapiens OX=9606 GN=OR5M9 PE=3 SV=1

>sp|Q8NGG0|OR8J3_HUMAN Olfactory receptor 8J3 OS=Homo sapiens OX=9606 GN=OR8J3 PE=3 SV=1

>sp|P59025|RTP1_HUMAN Receptor-transporting protein 1 OS=Homo sapiens OX=9606 GN=RTP1 PE=2 SV=2

>sp|Q8VFD3|OL484_MOUSE Olfactory receptor 484 OS=Mus musculus OX=10090 GN=Olfr484 PE=3 SV=1

>sp|Q8VGS1|O1038_MOUSE Olfactory receptor 1038 OS=Mus musculus OX=10090 GN=Olfr1038 PE=3 SV=1

>sp|Q8VGI5|OL481_MOUSE Olfactory receptor 481 OS=Mus musculus OX=10090 GN=Olfr481 PE=3 SV=1

>sp|Q9TU86|OR1G1_GORGO Olfactory receptor 1G1 OS=Gorilla gorilla gorilla OX=9595 GN=OR1G1 PE=3 SV=1

>sp|Q8NGI7|O10V1_HUMAN Olfactory receptor 10V1 OS=Homo sapiens OX=9606 GN=OR10V1 PE=3 SV=3

>sp|Q8NGP9|O5AR1_HUMAN Olfactory receptor 5AR1 OS=Homo sapiens OX=9606 GN=OR5AR1 PE=3 SV=1

>sp|Q8VG09|OL502_MOUSE Olfactory receptor 502 OS=Mus musculus OX=10090 GN=Olfr502 PE=2 SV=1

>sp|Q8VEX5|OL186_MOUSE Olfactory receptor 186 OS=Mus musculus OX=10090 GN=Olfr186 PE=3 SV=1

>sp|Q8VGK5|OLF50_MOUSE Olfactory receptor 50 OS=Mus musculus OX=10090 GN=Olfr50 PE=2 SV=1

>sp|Q8NGP6|OR5M8_HUMAN Olfactory receptor 5M8 OS=Homo sapiens OX=9606 GN=OR5M8 PE=2 SV=1

>sp|Q96RD0|OR8B2_HUMAN Olfactory receptor 8B2 OS=Homo sapiens OX=9606 GN=OR8B2 PE=3 SV=3

>sp|A6NL26|OR5BL_HUMAN Olfactory receptor 5B21 OS=Homo sapiens OX=9606 GN=OR5B21 PE=3 SV=1

>sp|Q80XE1|RIC8B_MOUSE Synembryn-B OS=Mus musculus OX=10090 GN=Ric8b PE=1 SV=2

>sp|Q7TR96|O1013_MOUSE Olfactory receptor 1013 OS=Mus musculus OX=10090 GN=Olfr1013 PE=3 SV=1

>sp|Q8VF65|OL470_MOUSE Olfactory receptor 470 OS=Mus musculus OX=10090 GN=Olfr470 PE=3 SV=1

>sp|Q8VG13|OL507_MOUSE Olfactory receptor 507 OS=Mus musculus OX=10090 GN=Olfr507 PE=3 SV=1

>sp|Q8NH18|OR5J2_HUMAN Olfactory receptor 5J2 OS=Homo sapiens OX=9606 GN=OR5J2 PE=3 SV=1

>sp|O95371|OR2C1_HUMAN Olfactory receptor 2C1 OS=Homo sapiens OX=9606 GN=OR2C1 PE=2 SV=3

>sp|Q15620|OR8B8_HUMAN Olfactory receptor 8B8 OS=Homo sapiens OX=9606 GN=OR8B8 PE=2 SV=2

>sp|Q8NGQ4|O10Q1_HUMAN Olfactory receptor 10Q1 OS=Homo sapiens OX=9606 GN=OR10Q1 PE=2 SV=1

>sp|Q8NGZ2|O14K1_HUMAN Olfactory receptor 14K1 OS=Homo sapiens OX=9606 GN=OR14K1 PE=3 SV=2

>sp|P23268|O1082_RAT Olfactory receptor 1082 OS=Rattus norvegicus OX=10116 GN=Olr1082 PE=2 SV=1

>sp|Q8VG07|OL494_MOUSE Olfactory receptor 494 OS=Mus musculus OX=10090 GN=Olfr494 PE=3 SV=1

>sp|Q8VEW2|OL498_MOUSE Olfactory receptor 498 OS=Mus musculus OX=10090 GN=Olfr498 PE=3 SV=1

>sp|Q96R09|OR5B2_HUMAN Olfactory receptor 5B2 OS=Homo sapiens OX=9606 GN=OR5B2 PE=2 SV=3

>sp|P34175|PBP_HYACE Pheromone-binding protein (Fragment) OS=Hyalophora cecropia OX=7123 PE=1 SV=1

>sp|Q8NGE7|OR9K2_HUMAN Olfactory receptor 9K2 OS=Homo sapiens OX=9606 GN=OR9K2 PE=2 SV=2

>sp|Q8VG06|OL491_MOUSE Olfactory receptor 491 OS=Mus musculus OX=10090 GN=Olfr491 PE=3 SV=1

>sp|Q8VEW6|OL510_MOUSE Olfactory receptor 510 OS=Mus musculus OX=10090 GN=Olfr510 PE=3 SV=1

>sp|Q9TUA8|OR1D2_PANTR Olfactory receptor 1D2 OS=Pan troglodytes OX=9598 GN=OR1D2 PE=3 SV=1

>sp|Q17077|PBP1_ANTPE Pheromone-binding protein 1 OS=Antheraea pernyi OX=7119 PE=2 SV=1

>sp|Q8NGX0|O11L1_HUMAN Olfactory receptor 11L1 OS=Homo sapiens OX=9606 GN=OR11L1 PE=2 SV=1

>sp|Q9NZP5|O5AC2_HUMAN Olfactory receptor 5AC2 OS=Homo sapiens OX=9606 GN=OR5AC2 PE=3 SV=2

>sp|Q8VFD2|OL490_MOUSE Olfactory receptor 490 OS=Mus musculus OX=10090 GN=Olfr490 PE=3 SV=1

>sp|Q8NGL1|OR5DI_HUMAN Olfactory receptor 5D18 OS=Homo sapiens OX=9606 GN=OR5D18 PE=2 SV=1

>sp|Q8NGJ0|OR5A1_HUMAN Olfactory receptor 5A1 OS=Homo sapiens OX=9606 GN=OR5A1 PE=2 SV=1

>sp|Q8N127|O5AS1_HUMAN Olfactory receptor 5AS1 OS=Homo sapiens OX=9606 GN=OR5AS1 PE=1 SV=1

>sp|Q95154|OLF1_CANLF Olfactory receptor-like protein OLF1 OS=Canis lupus familiaris OX=9615 PE=3 SV=1

>sp|Q6IEU7|OR5MA_HUMAN Olfactory receptor 5M10 OS=Homo sapiens OX=9606 GN=OR5M10 PE=2 SV=1

>sp|Q8NGE9|OR9Q2_HUMAN Olfactory receptor 9Q2 OS=Homo sapiens OX=9606 GN=OR9Q2 PE=2 SV=1

>sp|Q95155|OLF2_CANLF Olfactory receptor-like protein OLF2 OS=Canis lupus familiaris OX=9615 PE=3 SV=1

>sp|Q8VG08|OL497_MOUSE Olfactory receptor 497 OS=Mus musculus OX=10090 GN=Olfr497 PE=3 SV=1

>sp|Q8VGI6|OL477_MOUSE Olfactory receptor 477 OS=Mus musculus OX=10090 GN=Olfr477 PE=3 SV=1

>sp|P37069|OLF3_CHICK Olfactory receptor-like protein COR3 OS=Gallus gallus OX=9031 GN=COR3 PE=3 SV=1

>sp|Q8NGG7|OR8A1_HUMAN Olfactory receptor 8A1 OS=Homo sapiens OX=9606 GN=OR8A1 PE=2 SV=2

>sp|Q9QXU1|MSP_MESAU Male-specific submandibular salivary gland protein OS=Mesocricetus auratus OX=10036 GN=MSP PE=1 SV=2

>sp|Q99MG7|FLP_MESAU Female-specific lacrimal gland protein OS=Mesocricetus auratus OX=10036 GN=FLP PE=1 SV=1

>sp|Q8VF66|OL469_MOUSE Olfactory receptor 469 OS=Mus musculus OX=10090 GN=Olfr469 PE=3 SV=1

>sp|P37071|OLF5_CHICK Olfactory receptor-like protein COR5 OS=Gallus gallus OX=9031 GN=COR5 PE=3 SV=1

>sp|P37067|OLF1_CHICK Olfactory receptor-like protein COR1 OS=Gallus gallus OX=9031 GN=COR1 PE=3 SV=1

>sp|P0C7N1|OR8U8_HUMAN Olfactory receptor 8U8 OS=Homo sapiens OX=9606 GN=OR8U8 PE=3 SV=1

>sp|Q8NHB8|OR5K2_HUMAN Olfactory receptor 5K2 OS=Homo sapiens OX=9606 GN=OR5K2 PE=1 SV=3

>sp|Q8NGG8|OR8B3_HUMAN Olfactory receptor 8B3 OS=Homo sapiens OX=9606 GN=OR8B3 PE=3 SV=3

>sp|Q8N162|OR8H2_HUMAN Olfactory receptor 8H2 OS=Homo sapiens OX=9606 GN=OR8H2 PE=3 SV=1

>sp|Q8NGG5|OR8K1_HUMAN Olfactory receptor 8K1 OS=Homo sapiens OX=9606 GN=OR8K1 PE=2 SV=1

>sp|Q8C8C1|RTP1_MOUSE Receptor-transporting protein 1 OS=Mus musculus OX=10090 GN=Rtp1 PE=1 SV=1

>sp|Q5QGU6|RTP3_MOUSE Receptor-transporting protein 3 OS=Mus musculus OX=10090 GN=Rtp3 PE=1 SV=2

>sp|Q8VFB9|OL183_MOUSE Olfactory receptor 183 OS=Mus musculus OX=10090 GN=Olfr183 PE=3 SV=1

>sp|Q98914|OLF9_CHICK Olfactory receptor-like protein COR9 (Fragment) OS=Gallus gallus OX=9031 GN=COR9 PE=3 SV=1

>sp|P0C626|OR5G3_HUMAN Olfactory receptor 5G3 OS=Homo sapiens OX=9606 GN=OR5G3 PE=3 SV=1

>sp|Q96R08|OR5BC_HUMAN Olfactory receptor 5B12 OS=Homo sapiens OX=9606 GN=OR5B12 PE=2 SV=2

>sp|Q27388|PBP_HELVI Pheromone-binding protein OS=Heliothis virescens OX=7102 PE=2 SV=2

>sp|P23267|OL287_RAT Olfactory receptor 287 OS=Rattus norvegicus OX=10116 GN=Olr287 PE=2 SV=1

>sp|P34178|PBP_ORGPS Pheromone-binding protein (Fragment) OS=Orgyia pseudotsugata OX=33414 PE=1 SV=1

>sp|Q8NHC6|O14L1_HUMAN Putative olfactory receptor 14L1 OS=Homo sapiens OX=9606 GN=OR14L1P PE=5 SV=1

>sp|Q8NGF4|O5AP2_HUMAN Olfactory receptor 5AP2 OS=Homo sapiens OX=9606 GN=OR5AP2 PE=2 SV=1

>sp|Q8VFV4|O1440_MOUSE Olfactory receptor 1440 OS=Mus musculus OX=10090 GN=Olfr1440 PE=2 SV=1

>sp|Q8NGR4|OR5C1_HUMAN Olfactory receptor 5C1 OS=Homo sapiens OX=9606 GN=OR5C1 PE=2 SV=1

>sp|Q8NGM9|OR8D4_HUMAN Olfactory receptor 8D4 OS=Homo sapiens OX=9606 GN=OR8D4 PE=2 SV=1

>sp|Q9NH75|VA4_SOLGE Venom allergen 4 OS=Solenopsis geminata OX=121131 PE=1 SV=1

>sp|Q27017|B1_TENMO B1 protein (Fragment) OS=Tenebrio molitor OX=7067 PE=2 SV=1
